# Standardized assessment of bone micromorphometry around teeth following orthodontic tooth movement: A µCT split-mouth study in mice

**DOI:** 10.1007/s00056-021-00336-9

**Published:** 2021-08-03

**Authors:** Viktoria Trelenberg-Stoll, Michael Wolf, Caroline Busch, Dieter Drescher, Kathrin Becker

**Affiliations:** 1 Department of Oral Surgery, Universitätsklinikum Düsseldorf, Düsseldorf, Germany; 2 Department of Orthodontics, Universitätsklinikum RWTH Aachen, Aachen, Germany; 3 Department of Orthodontics, Universitätsklinikum Düsseldorf, 40225 Düsseldorf, Germany

**Keywords:** Watershed algorithm, Periodontal bone analysis, Microcomputed tomography, Tooth protraction, Volumetric analysis, Wasserscheidentransformation, Parodontale Knochenanalyse, Mikro-Computertomographie, Zahnbewegung, Volumetrische Analyse

## Abstract

**Purpose:**

Volumetric quantitative analyses of bone micromorphometry changes following orthodontic tooth movements are hardly standardizable. The present study aimed at validating and applying a novel microcomputed tomography (CT)-based approach that enables the segmentation of teeth and definition of a standardized volume of interest (VOI) around the roots to assess local bone micromorphometry.

**Methods:**

The jaws of 3 untreated and 14 orthodontically treated mice (protraction of the upper right molar for 11 days with 0.5 N; untreated left upper molar) were scanned with a micro-CT. The first molars and the alveolar bone were segmented, and a standardized VOI was defined around the teeth. The bone volume per total volume (BV/TV) was assessed within the VOI, and BV/TV values were compared between contralateral sites in both untreated (method validation) and treated animals (method application).

**Results:**

The intraclass correlation coefficient of 0.99 revealed high reliability of the method. In the untreated animals, Bland–Altman analysis confirmed comparable BV/TV fractions (mean difference: −1.93, critical difference: 1.91, Wilcoxon: *p* = 0.03). In the orthodontically treated animals, BV/TV values were significantly lower at the test compared to the control site (test: 33.23% ± 5.74%, control: 41.33% ± 4.91%, Wilcoxon: *p* < 0.001).

**Conclusion:**

Within the limits of the study, the novel approach demonstrated the applicability to evaluate bone micromorphometry around teeth subjected to orthodontic treatment.

## Abstract (de)

**Hintergrund:**

Volumetrische quantitative Analysen von Veränderungen der Knochenmikrostruktur nach kieferorthopädischen Zahnbewegungen sind schwer standardisierbar. Ziel der vorliegenden Studie war die Validierung und Anwendung eines neuartigen mikro-computertomographie(µ-CT)-basierten Ansatzes, der die Segmentierung von Zähnen und die Definition eines standardisierten „volume of interest“ (VOI) um die Wurzeln herum ermöglicht, um die lokale Knochenmikrostruktur zu beurteilen.

**Methoden:**

Die Kiefer von 3 unbehandelten und 14 kieferorthopädisch behandelten Mäusen (Protraktion des oberen rechten Molaren für 11 Tage mit 0,5 N; unbehandelter linker oberer Molar) wurden mit einem Mikro-CT gescannt. Die ersten Molaren und der Alveolarknochen wurden segmentiert und ein standardisiertes VOI um die Zähne definiert. Das Knochenvolumen pro Gesamtvolumen (BV/TV) wurde innerhalb des VOIs bestimmt. Die BV/TV-Werte wurden zwischen den kontralateralen Seiten sowohl bei unbehandelten (Methodenvalidierung) als auch bei kieferorthopädisch behandelten Tieren (Methodenanwendung) verglichen.

**Ergebnisse:**

Der Intraklassenkorrelationskoeffizient von 0,99 zeigte eine hohe Reliabilität der Methode. Bei den unbehandelten Tieren bestätigte die Bland-Altman-Analyse vergleichbare BV/TV-Werte (mittlere Differenz: −1,93, kritische Differenz: 1,91, Wilcoxon-Test: *p* = 0,03). Bei den kieferorthopädisch behandelten Tieren waren die BV/TV-Werte in der Test- im Vergleich zur Kontrollgruppe signifikant niedriger (Test: 33,23 % ± 5,74 %, Kontrolle: 41,33 % ± 4,91 %, Wilcoxon-Test: *p* < 0,001).

**Schlussfolgerung:**

Im Rahmen der Studie zeigte der neuartige Ansatz die Anwendbarkeit zur Bewertung der Knochenmikrostruktur um Zahnwurzeln von Molaren, die einer kieferorthopädischen Behandlung unterzogen wurden.

## Introduction

Bone regeneration around teeth is a consequence of complex bone remodeling which involves a balance of resorption of mineralized bone and formation of new bone matrix [1–3]. Evaluation and quantification of bone micromorphometry around teeth can be of interest in the orthodontic field, but also to assess bone regeneration at periodontally compromised teeth [4]. For this purpose, histological examinations were frequently employed [5–9]. Major drawbacks of histological approaches, however, are the limitation to two dimensions and information loss during undecalcified sectioning. In addition, bone microstructure may largely vary with respect to the cutting position. Eventually, most histological analyses are limited to end-point analyses [10–12].

In contrast, microcomputed tomography (micro-CT) is a nondestructive alternative overcoming the above-mentioned limitations. It provides high-resolution volumetric images and enables three-dimensional (3D) analyses of bone microstructural properties [13–15]. For small animals, the dynamics of bone remodeling can be studied even longitudinally by means of in vivo micro-CT. If only end-point analyses are possible, corresponding contralateral sites can be compared instead [16, 17].

To perform 3D quantitative and qualitative analyses of hard tissue around teeth, segmentation of the alveolar bone and definition of standardized volumes of interest (VOIs) is mandatory. This step can be challenging when histograms from bone, cement and dentin overlap [18]. Therefore, the majority of previous micro-CT studies performed linear measurements in two-dimensional (2D) slices around teeth or defined a rectangular VOIs between tooth roots to analyze the alveolar bone microstructural properties [19, 20]. To the best knowledge of the authors, no methods have been reported for standardized automated analyses of the alveolar bone around teeth/periodontal ligament space.

The marker-based Watershed algorithm (WS) has been described in the literature as a tool to segment tissues with overlapping histograms in volumetric radiographic images [21, 22]. After placement of different markers at each anatomical structure, they will be enlarged until reaching the closest edge. Eventually, a labeled image is created that can be used for standardized definitions of VOIs and microstructural analyses.

Therefore, the present study aimed at presenting and validating a novel WS-based method for automated and standardized assessment of bone micromorphometry around tooth roots following orthodontic tooth movement in split-mouth preclinical animal studies.

## Materials and methods

### Animals

**Fig. 1:**
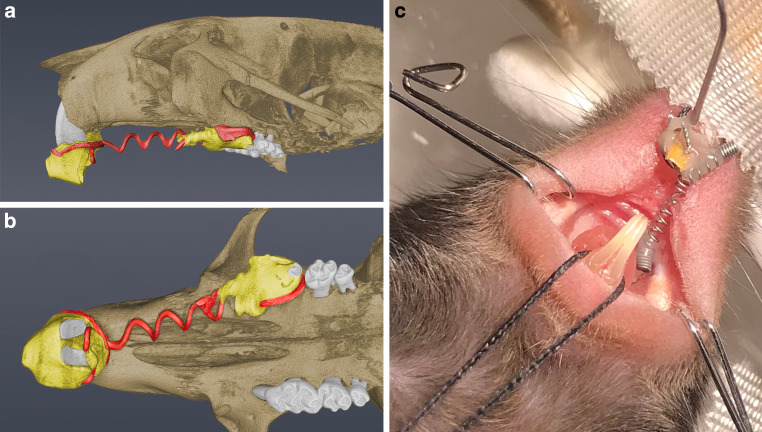
Volumetric rendering of a microcomputed tomography (CT) scan showing the orthodontic appliance from sagittal (**a**) and occlusal (**b**) view. The activated nickel–titanium coil spring (force: 0.5 N) is *red*; the composite is *yellow*. **c** Clinical photograph showing the installation of the orthodontic appliance between the left incisor and upper left first molar Die volumetrische Darstellung eines Mikrocomputertomographie(µ-CT)-Scans zeigt die kieferorthopädische Apparatur aus sagittaler (**a**) und okklusaler (**b**) Ansicht. Die aktivierte Nickel-Titan-Feder (Kraft: 0,5 N) ist *rot* eingefärbt, das Komposit *gelb*. **c** Das klinische Foto veranschaulicht die Insertion der kieferorthopädischen Apparatur zwischen dem linken Schneidezahn und dem oberen linken ersten Molaren

For method validation (Method part), 3 female mice (BALB/c strain, age 5.2–5.6 months) that did not retrieve any orthodontic treatment were included. For method application (Application part), assessment of the bone volume fraction (BV/TV) was carried out in 14 mice (11 animals: *Enpp1*^*asj−2J*^ (BALB/cJ-*Enpp1*^asj−2J^/GrsrJ) deficient, 3 animals: littermate wild type; 9 females, 5 males). In these animals, a stretched 0.012-inch nickel–titanium closed coil spring (force: 0.5 N) was attached between the left upper first molar and the incisors for 11 days (Fig. 1). According to the previously published protocols [20, 23], the animals had an age of 60 days when orthodontic treatment was initiated. A 2D analysis of micro-CT scans (distance measurement between protracted molar and second molar, vertical bone loss, periodontal ligament space width), immunohistochemistry and histological findings have been reported previously for the same animals (and additional animals not included in the present study due to lacking micro-CT scans) [20]. All experiments were conducted in accordance with the appropriate animal care committees and law (Central institution for animal research and scientific research protection tasks, University hospital of Düsseldorf, Germany. National Institute of Arthritis and Musculoskeletal and Ski Diseases [NIAMS] Animal Care and Use Committee, reference number: A016-12-09).

### Microcomputed tomographic analysis

#### Method part

After sacrificing the animals, the skulls were harvested and the jaws were scanned with a micro-CT (Viva CT 80; Scanco Medical AG, Brüttisellen, Switzerland) operated at 70 kVp, 114 μA, 8 W, 31.9 mm FOV, 1500 projections, and an integration time of 500 ms. The data sets were reconstructed into three-dimensional (3D) volumes with an isotropic nominal resolution of 10.4 μm voxel size.

#### Application part

The samples were scanned with a micro-CT 50 (Micro-CT 50; Scanco Medical AG, Brüttisellen, Switzerland) operated at 70 kVp, 76 µA, 300–900 ms integration time and 9–10 µm voxel size.

### Image processing

Image processing was performed using Amira software (v6.5, FEI Visualization Science Group, Burlington, MA, USA) by a trained investigator (V. T.-S.) and validated by another author (C. B.). All steps for the Method part, which are described below, were performed in triplicate for validation purposes.

#### Bone and tooth segmentation

##### Method part

**Fig. 2:**
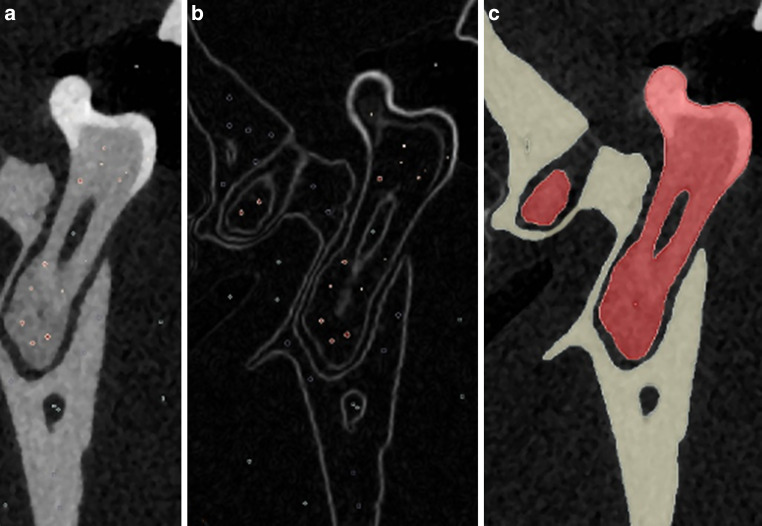
The three steps of the marker-based Watershed segmentation procedure are shown. Manually placed seed points label each tissue that is going to be segmented. The algorithm enlarges each label until it touches the edges of the respective material. **a** Placement of the seed points (materials: teeth, air, and bone). **b** Application of an edge filter (i.e., creation of the gradient image), seed points still have their initial size. **c** Growing of the seed points to the boundaries of the gradient image. The seed points now label the respective materials. Color convention: Bone (*beige*), tooth (*red*), air (*black*) Die 3 Schritte des markerbasierten Wasserscheidentransformationssegmentierungsverfahrens. Manuell platzierte Marker definieren jedes zu segmentierende Gewebe. Der Algorithmus vergrößert jede Markierung, bis sie die Kanten des jeweiligen Materials berührt. **a** Platzierung der Marker (Materialien: Zähne, Luft und Knochen). **b** Anwendung eines Kantenfilters (zur Erstellung des Gradientenbildes), die Marker haben noch ihre Anfangsgröße. **c** Wachsen der Marker an die Grenzen des Gradientenbildes. Die Marker kennzeichnen nun die jeweiligen Materialien. Farbkonvention: Knochen (*beige*), Zahn (*rot*), Luft (*schwarz*)

All first molars and the surrounding bone were segmented using a marker-based Watershed algorithm (WS). In detail, a median filter (*n* = 3 iterations) was applied, and a gradient image was computed using the Sobel operator for edge detection. Then, the following classification labels were defined per jaw and quadrant: first molar, bone, and air. For each label, seed points were located manually by marking voxels belonging to the respective tissue. The seed points were subject to automated growing towards the edges of the gradient image (Fig. 2).

##### Application part

The described WS method was used to segment the first upper molars, the adjacent bone, and the background (air).

#### Alignment of bone and molars to the axes of the coordinate system

##### Method part

Each segmented molar was aligned such that the normal vector of the plane containing the cementoenamel junction (CEJ) coincided with the z‑axis from the Euclidean coordinate system. For this purpose, three coordinate vectors from the CEJ plane were selected manually and aligned to the xy-plane by means of a principal component analysis which was performed in Matlab (Matlab R2015a 64-bit, The Mathworks Inc., MA, USA). After aligning the segmented molar, the calculated translation and rotation coordinates were transferred to the segmented bone tissue.

##### Application part

In the application part, the alignment was applied to the upper molars and the alveolar bone only.

#### Separating the molar roots

##### Method part

**Fig. 3:**
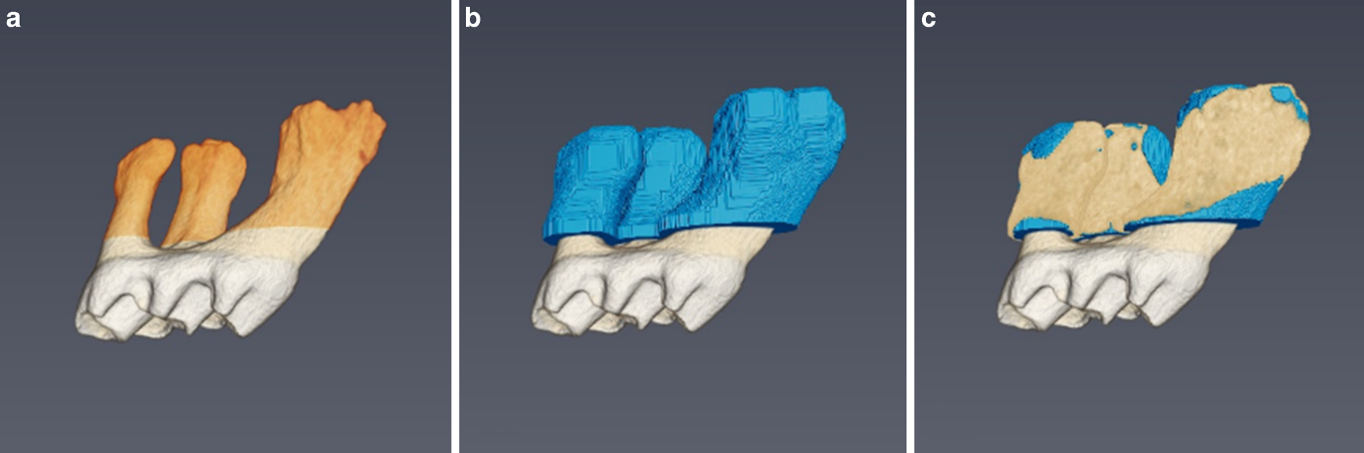
Definition of a standardized volume of interest (VOI) around the tooth roots for calculation of the bone fraction (BV/TV) values. **a** Definition of the height of the VOI (*orange*). **b** Definition of the width (color: *blue*, distance of 100 μm). **c** Visualization of the entire volume of interest (VOI) showing also the fraction of periodontal bone. Color convention: Periodontal bone (*beige*), nondecalcified space of VOI (*blue*) Definition eines standardisierten „volume of interest“ (VOI) um die Zahnwurzeln zur Berechnung der Knochenfraktion (BV/TV). **a** Veranschaulichung der VOI-Höhe (*orange*). **b** Veranschaulichung der Breite (Farbe: *blau*, Abstand von 100 μm). **c** Visualisierung des gesamten VOI einschließlich des parodontalen Knochenanteils. Farbkonvention: parodontaler Knochen (*beige*), nichtentkalkter Bereich des VOI (*blau*)

After alignment of the CEJ of each upper and lower first molar to the xy-plane of the coordinate system, roots were separated from the teeth by cropping them at 30 voxels apical to the CEJ (Fig. 3a).

##### Applaction part

After alignment of the CEJ from each upper first molar to the xy-plane of the coordinate system, roots were separated from the teeth by cropping them at 30 voxels apical to the CEJ.

#### Bone volumes

##### Method part and application part

After separation of the molar roots, standardized VOIs were created as follows: All “holes” (corresponding to the root canals) were filled virtually, and a dilation filter was applied to increase the size of the roots by 100 μm. The originally sized roots were then subtracted from the enlarged ones to retrieve the standardized VOI (Fig. 3b). To assess the bone volume per tissue volume (BV/TV) within VOI, the amount of calcified within VOI was quantified (Fig. 3c).

### Statistical analysis

The statistical analysis was performed using the software program R [24].

#### Method part

Reliability of the segmentation procedures was analyzed by computing the respective intraclass correlation coefficients (ICC). For descriptive purposes, the means and standard deviations were computed. To assess agreement among repeated measurements, Bland–Altman analyses were employed [25]. Contralateral BV/TV values were compared using the Wilcoxon signed rank test. Results were considered significant at *p* < 0.05.

#### Application part

For descriptive purposes, the means and standard deviations as well as boxplots were created. The Wilcoxon signed rank test was used to assess differences in BV/TV values between the test and control sites. Results were considered significant at *p* < 0.05.

## Results

### Method part

The presented approach allowed to segment teeth and bone tissue, and to define standardized volumes of interest (VOI) around teeth for micromorphometrical analyses of alveolar bone.

#### Reliability of the method

Repetition of all procedures (image segmentation, definition of the VOI, calculation of BV/TV values) in triplicate confirmed the high reliability of the method. The respective intraclass correlation coefficient (ICC) was 0.99.

#### Comparability of left and right periodontal bone volumes

The overall BV/TV values amounted to 62.19% ± 1.80%. In the upper jaw, slightly lower values were identified (61.41% ± 1.73%) compared to the lower jaw (63.05% ± 1.44%).

**Fig. 4:**
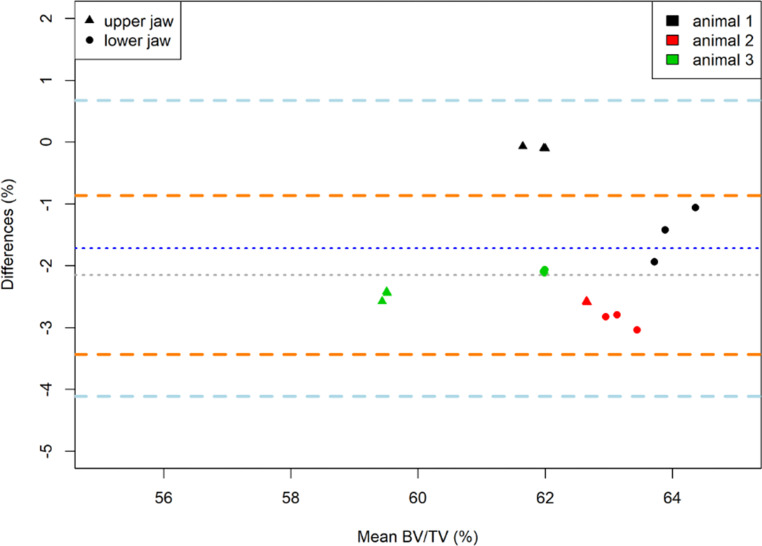
Bland–Altman plots showing the BV/TV values from repeated measurements in the upper and lower jaw as well as the right and left side in the untreated animals (Method part). The difference of repeated measurements in the upper jaw were low and amounted to −1.72%, agreement limits: 0.68% and −4.11% (upper jaw) and −2.15%, agreement limits −0.86% and −3.43% (lower jaw) Der Bland-Altman-Plot zeigt die BV/TV-Werte aus wiederholten Messungen im Ober- und Unterkiefer sowie der rechten und linken Seite bei den unbehandelten Tieren (Methodenteil). Die Differenz der Wiederholungsmessungen im Oberkiefer war mit −1,72% gering, Übereinstimmungsgrenzen: 0,68% und −4,11% (Oberkiefer), im Vergleich zum Unterkiefer −2,15%, Übereinstimmungsgrenzen −0,86% und −3,43% (Unterkiefer)

Bland–Altman analyses confirmed high comparability of BV/TV around the left and right upper (mean difference 1.72%), and lower molars (mean difference: 2.15%) (Fig. 4). This finding is in line with the Wilcoxon signed rank test which did not reveal any significant difference in BV/TV values between the contralateral sites (upper jaw: *p* = 0.25; lower jaw: *p* = 0.25).

### Application part

**Fig. 5:**
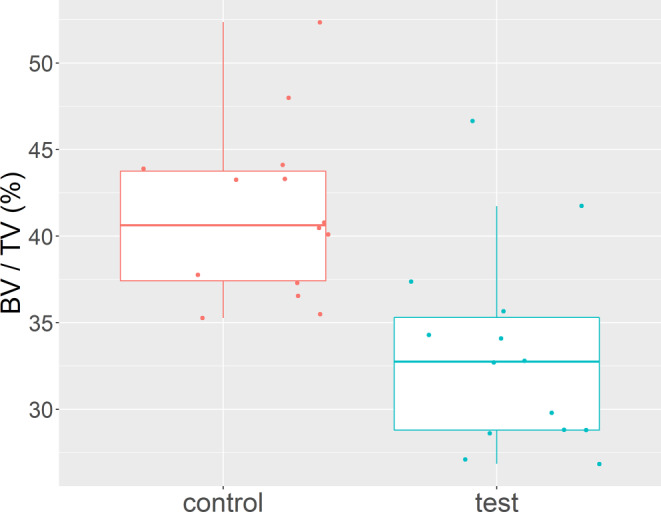
Boxplot showing the differences in BV/TV values between the control (*left*) and the orthodontically treated test side (*right*). BV/TV values were significantly lower after 11 days of molar protraction (BV/TV: *p* = 0.001, test: 33.23 ± 5.74%, control 41.33 ± 4.91%) Der Boxplot stellt die BV/TV-Wert-Unterschiede der Kontrolle (*links*) und der kieferorthopädisch behandelten Testgruppe (*rechts*) dar. Die BV/TV-Werte waren nach 11 Tagen Molarenprotraktion signifikant niedriger (BV/TV: *p* = 0,001, Test: 33,23 ± 5,74%, Kontrolle 41,33 ± 4,91%)

**Fig. 6:**
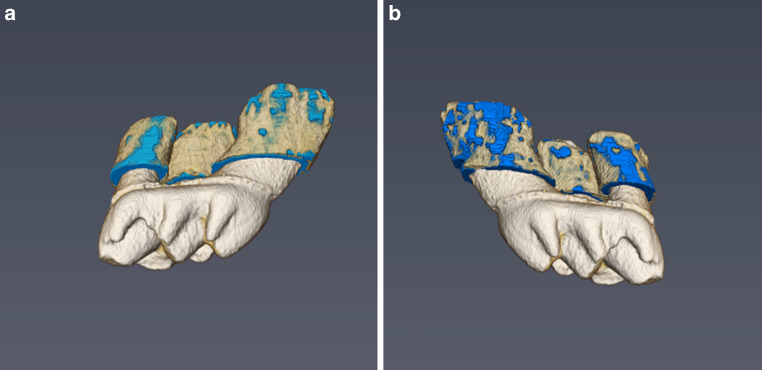
Visualization of an unaffected (**a**) and orthodontically protracted (**b**) molar with its surrounding periodontal bone tissue. Lower amounts of calcified tissue can be seen around the mesial root of the protracted molar Darstellung eines unbewegten (**a**) und eines kieferorthopädisch protrahierten (**b**) Molaren mit dem umgebenden parodontalen Knochengewebe. Um die mesiale Wurzel des protrahierten Molaren sind geringere Mengen an kalzifiziertem Gewebe zu erkennen

The overall BV/TV values at the protracted upper right molars amounted to 33.23% ± 5.74%, whereas significantly higher values were identified at the untreated left side, i.e., 41.33% ± 4.91% (*p* = 0.001; Fig. 5). Qualitative visual examination also revealed a more pronounced loss of calcified tissue around the mesial root, whereas minor differences were observed around the remaining two roots (Fig. 6).

## Discussion

Microstructural analyses of bone in conventional histology are limited to two dimensions and crucially depend on the selected cutting position. Whereas histology provides valuable information on the cell level, micro-CT has been reported to be an accurate and complementary technique to assess bone remodeling in all three dimensions [11, 13]. In case of overlapping histograms from bone and teeth, bone segmentation can be challenging and may require advanced methodologies. To allow quantitative comparisons, standardized definition of a volume of interest (VOI) is required. In previous studies, linear 2D measurements and cubic VOIs have been frequently applied. However, no automated and standardized approaches have been reported for reliable segmentation and definition of standardized VOIs around tooth roots. Therefore, the present study aimed at presenting and validating a method for automated and standardized assessment of bone micromorphometry around tooth roots, and to apply the novel method to micro-CT scans from a preclinical animal study performing orthodontic tooth protraction in mice. The respective 2D measurements have been published previously [20].

The intraclass correlation coefficient amounted to 0.99 and confirmed the high reliability of the novel approach.

When comparing BV/TV values between contralateral sites in the untreated animals (Method part), Bland–Altman analyses revealed negligible differences of 1.72% in the upper, and of 2.15% in the lower jaw. Nevertheless, it has to be noted that BV/TV values were slightly more heterogeneous in the lower jaw. The differences between animals were greater than differences between contralateral sites and amounted to 3.17% ± 1.23% in the upper jaw and 2.00% ± 0.71% in the lower jaw.

Application of the novel method (Application part) revealed significantly lower BV/TV values at protracted molars compared to the contralateral control sites, probably resulting from bone resorption in the pressure zones, and less mineralized newly formed bone at the tension zones.

Undecalcified regions were mostly found in proximity to the tooth root and decreased towards the borders of the 100 µm VOI, suggesting that bone resorption was highest in close proximity to the roots but still present in the selected region. In addition, bone resorption was highest at the mesial roots which is in line with a recent 3D analysis showing that intrusion and mesial–palatal tipping was the most common movement of the protracted teeth [26].

Nonetheless, definition of a reliable VOI is challenging. It has to be large enough to be representative, whereas on the other hand, it must not exceed the jawbone. Furthermore, it should be limited to the areas in which bone remodeling occurs. In the present study, a diameter of 100 μm was found to be optimal, as this was the maximum possible size giving the boundaries of the jawbone. The Application part confirmed that bone remodeling occurred in the VOIs, which demonstrated that the analyzed regions were not too small.

When comparing the present findings with the previously published data, it has to be noted that no significant differences in vertical bone loss could be found in the previously published 2D measurements [20]. In contrast, the present analysis revealed a significant decrease of BV/TV at the test site. In addition, it revealed that bone loss was most pronounced at the mesial root and decreased towards periphery. Hence, we believe that this novel approach is a valuable tool to better understand volumetric changes in bone micromorphology following orthodontic tooth movements.

Limitations of the present investigation include the lack of longitudinal data; thus, different animals were used for method validation, and the comparison of the test and control sites after 11 days of molar protraction. Furthermore, the study design did not allow differentiation of whether the minor BV/TV differences between animals from the Method part resulted from differences in genetic background, age, or were related to the orthodontic appliance which might have impaired food intake. In addition, analyses were limited to assessment of BV/TV values. Additional parameters such as trabecular thickness, bone mineral density, bone surface area or trabecular spacing may be calculated in future studies utilizing the presented approach to understand the impact of genetic disorders, metabolic diseases or drug intake on bone remodeling during orthodontic treatments.

## Conclusion

Within its limitations, the present study provides a novel approach to assess bone micromorphometry in micro-CT scans within a standardized volume of interest around murine teeth. It confirmed high agreement of BV/TV values between contralateral sites in untreated animals. Application of the method to animals subjected to orthodontic tooth protraction confirmed significant reduction of mineral content at the test site which was most pronounced at the mesial root.
